# Spirits

**Published:** 1882-03

**Authors:** 


### SPIRITS.

It is worse than useless, for men of learning and repute to reiterate their impatient denials of palpable facts which all of us can see for ourselves. There is such a thing as glorying in one's shame. Intelligent persons have taken a pride in exhibiting their utter incredulity as to the facts connected with so called spiritualism, and have exhibited more incredulity in maintaining incredible hypotheses than does the most uncultivated mind in giving as a reason that the world does not turn round, the soup would otherwise fall into the fire.

However refined, and educated and talented a man may be, the moment he is supposed to advocate "*Spiritualism*" he is voted a fool, going crazy, lost his senses, with other epithets equally complimentary.

There are thousands of facts which have been hooted at until explanations have demonstrated their naturalness, and anon we feel assured that they could not have been otherwise. It creates no surprise in us to see a dozen needles jump up from the table at the approach of the *Armature*, yet if you were to take a magnet in your hand and explain to an ignorant mind that it would pick up a needle from the table without touching it, he would be utterly incredulous until he saw the fact for himself, and in an instant incredulity is exchanged for a firm conviction, and an overwhelming awe. Were I to say to my readers that I had a substance, which if thrown into the water would dance until it was dead, would take fire and burn up, some of them would, even if they saw it with their own eyes, declare it was a trick, and would refuse to believe otherwise, until the nature of the substance was explained to them. Precisely thus is it with the facts in connection with the misnomer Spiritualism. The *Rubicon* of utter incredulity and confirmed conviction is—explanation, show the philosophy of the thing, teach how it is done—the cloud disperses, the fog scatters, the curtain is drawn up, and what but a single moment before was hated as a humbug, is fondled as a fact, is received with that loving welcome so familiar to those who *seek the truth in the love of it.*

It is the confounding fact with fancy, which has filled the world with the "*Spiritual*" folly. The theory of a truth is [illegible]

very different thing from the truth itself: but to deny a fact because the theory of it is absurd, is absurdity itself: and yet the mass of men of mind in this country, and in Europe too, have committed that very absurdity, and not a few men of note, more pious than profound, have pronounced "SPIRITUALISM," *an invention of the Devil.* An uncultivated young girl from the country exhibits certain phenomena which we see and hear; she says "*it's the spirits,*" and we turn around and exclaim with most undignified impatience, "*it's all nonsense.*" Is it not wonderful that an educated mind should have so little astuteness as to confound theory and fact together and brand them both as a falsehood in spite of the evidence of his eyes and ears, instead of separating them, and pronouncing upon each, according to its individual merit. Verily, this is a new era in Æsthetics, the race of analytical philosophers in mental science is becoming extinct and common sense obsolete.

The view which we take of Spiritualism is this. The facts are facts, but the "spirits," where are they? Echo answers *non est inventus*, out and gone, like Granger's eye.

The tables can rap and tap, and hobble about the room without collusion, will run over, by, against and through people if they don't get out of the way; a person may get on the table, and with no other agency than its being touched with the finger of a frail girl, that table will move about the room with the person on it; tables will rise up to meet the fingers; there, our *actual* knowledge ends; the next step, and we are in the land of the impalpable, and do but flounder in the fog.

Up to this time, many have risen with the exclamation, "*lo! here is the light,*" and often have we gone expectantly, only to be left in "darkness tangible," so that the prevailing feeling in our own mind, on hearing of a paper, or pamphlet, or book, or lecture, professing to *explain Spiritualism,* is to put our hand upon our pocket, as if there were an over busy finger about.

Our readers are indebted for the foregoing to our having received from a Poet clergyman, a communication of some twenty manuscript pages, which by the way we never expect to read. We have glanced over some of them, and gather the idea that he believes himself capable of *demonstrating.*

"1st. That table rappings, tappings, &c., are actual facts which can occur without collusion.

2d. "That the spirits of the departed have nothing to do with these phenomena."

As to the other points, without admiring the smoothness of his diction, or the perspicacity of his ideas, we give his own words:

*Seven propositions to be mathematically demonstrated before a Scientific committee in New York city :*

1st. That the muscular power is an elimination from the physical constitution.

2d. That the identical person is the agent and actor.

3d. That the physical and muscular power in voluntary mental attraction can be conveyed to and received by the one in *rapport.*

4th. That two inferior mediums can annul the physical effect and volition of the superior.

5th. That every organization has more or less of this physical, abstract, and volitic combination.

6. That contact is essential as a means of connexion, and yet may not be in advanced, peculiar and extraordinary developments.

7th. Human presence, perceptible by the slightest imperceptible attenuations.

"The above principles cover the whole ground of the tabular movement, and mathematical laws growing out of them, explain the identity of the Scientific or natural, and the alleged "*spirit power.*"

Often it is our involuntary exclamation "*whereunto will these things grow.*" Certain is it, *the end is not yet.* In the language of a Doctor, in "*sporadic cases*" the end has been infidelity, rationalism, dementia, madness, materialism, and what in our mind is worse than all, a sweeping rejection of the Holy Bible, as a divine revelation. So that in view of the whole subject, we say now to the masses, as we said just a year ago, *go not in the way of temptation. Touch not, taste not, handle not* this dangerous thing, for the touch of it has been to many spiritual death, a total uprooting of the faith of their fathers, leaving them

In endless mazes, LOST!

